# Oriented Boron Nitride in Calcium Alginate Matrix: A Sustainable Pathway to High-Efficiency Thermal Interface Materials

**DOI:** 10.3390/ma18122757

**Published:** 2025-06-12

**Authors:** Jiachen Sun, Dengfeng Shu, Fei Huang, Wenbo Qin, Wen Yue, Chengbiao Wang

**Affiliations:** 1 State Key Laboratory of Deep Earth Exploration and Imaging, School of Engineering and Technology, China University of Geosciences (Beijing), Beijing 100083, China; 15262596669@163.com (J.S.); huangfei66@outlook.com (F.H.); cbwang@cugb.edu.cn (C.W.); 2 School of Mechanical and Electrical Engineering, Hubei Three Gorges Polytech, Yichang 443000, China; shu_scaler@163.com; 3 HYMN Advance Materials Technology (Shenzhen), Shenzhen 518000, China; 4 Zhengzhou Institute, China University of Geosciences (Beijing), Zhengzhou 451283, China

**Keywords:** non-silicone thermal interface material, boron nitride, calcium alginate, orientation, thermal conductivity

## Abstract

With the rapid advancement of electronic devices toward higher frequencies, faster speeds, increased integration, and miniaturization, the resulting elevated operating temperatures pose significant challenges to the performance and longevity of electronic components. These developments have intensified the demand for high-performance thermal interface materials (TIMs). Conventional silicone rubber-based TIMs often suffer from silicone oil-bleeding and the volatilization of low-molecular-weight siloxanes under elevated temperatures and mechanical stress. The release of these volatile organic compounds can lead to their deposition on circuit boards and electronic components, causing signal interference or distortion in optical and electronic systems, ultimately compromising device functionality. Additionally, the intrinsic thermal conductivity of traditional TIMs is insufficient to meet the escalating demands for efficient heat dissipation. To overcome these limitations, this study introduces a novel, non-silicone TIM based on a calcium ion-crosslinked sodium alginate matrix, prepared via ion-exchange curing. This bio-derived polymer matrix serves as an environmentally benign alternative to silicone rubber. Furthermore, a brush-coating technique is employed to induce the oriented alignment of boron nitride (BN) fillers within the alginate matrix. Experimental characterization reveals that this aligned microstructure markedly enhances the thermal conductivity of the composite, achieving a value of 7.87 W·m^−1^·K^−1^. The resulting material also exhibits outstanding thermal and mechanical stability, with no observable leakage or condensate formation under high-temperature and high-pressure conditions. This work offers a new design paradigm for environmentally friendly, high-performance TIMs with considerable potential for advanced electronic and optoelectronic applications.

## 1. Introduction

Thermal interface materials (TIMs) are integral to the thermal management of electronic, energy, and optoelectronic systems. Their primary function is to fill micro-gaps between heat sources and heat sinks, thereby minimizing interfacial thermal resistance and facilitating efficient heat transfer from electronic components to heat dissipation structures, ensuring optimal operating temperatures [1,2,3]. With the rapid progression of technologies such as 5G communications, high-performance computing, and electric vehicles, electronic devices are increasingly characterized by elevated power densities and integration levels. This trend imposes stringent requirements on the thermal conductivity and long-term reliability of TIMs [4,5,6].

In response, considerable research efforts have been devoted to enhancing the thermal performance of TIMs through the incorporation of high-thermal-conductivity fillers—such as aluminum nitride, diamond, and carbon fibers—and by optimizing their distribution and alignment within the matrix [7,8,9,10]. However, in optoelectronic and laser communication systems, TIMs must also meet additional functional demands, including excellent electrical insulation, low dielectric constant, minimal matrix oil-bleeding under mechanical stress, and low volatility at elevated temperatures [11,12,13].

Conventional TIM matrices, particularly silicone rubber, often suffer from silicone oil oil-bleeding and the volatilization of low-molecular-weight siloxanes under thermal and mechanical stress. This phenomenon arises from the migration of unreacted siloxane segments to the material surface during thermal exposure [14,15,16]. The resulting condensates can accumulate on optical elements and electrical contacts, leading to optical interference, electrical instability, or even catastrophic device failure [17]. Although chemical modification strategies and stabilizer incorporation have been explored to mitigate these effects, they frequently compromise the material’s thermal and mechanical integrity [18,19]. Furthermore, commonly used thermally conductive fillers, such as diamond or aluminum nitride, either fall short in dielectric performance or introduce challenges in electrical insulation, limiting their application in highly sensitive optoelectronic environments.

To address these limitations, there is a critical need to identify alternative matrix systems that are inherently stable under high-temperature and high-pressure conditions while also being compatible with multifunctional, thermally conductive fillers. In this context, naturally derived polymers have garnered increasing attention due to their environmental sustainability and distinctive physicochemical properties [20]. Sodium alginate, a naturally occurring polysaccharide, readily forms a stable three-dimensional gel network through ion-exchange crosslinking with calcium ions—a process that is rapid, tunable, and operable at ambient conditions [21]. Calcium alginate gels possess desirable mechanical robustness, thermal stability, and biocompatibility, attributes that have led to their widespread use in biomedical and food-related applications [22,23,24]. Importantly, the polymeric structure of calcium alginate offers a promising matrix framework for the incorporation of thermally conductive fillers.

Among such fillers, boron nitride (BN)—commonly referred to as “white graphene”—has emerged as a highly promising candidate owing to its intrinsic high thermal conductivity and low dielectric constant. However, the strong anisotropy in its thermal conductivity, arising from its layered structure, necessitates the development of methods for achieving orientation-controlled alignment within the matrix.

Comprehensively, in the heat dissipation design of electronic components, high temperature is the main cause of the failure of electronic components and the acceleration of material aging. On the other hand, if the organic molecule precipitates, small molecules of organic molecule materials may precipitate, which may cause short circuits in circuits, the corrosion of components, or signal interference, seriously affecting the reliability and service life of electronic components. In the face of the trend of electronic products developing towards high integration and small sizes, TIMs that simultaneously possess high thermal conductivity and excellent chemical stability have the strong potential to meet the selection of the next generation of thermal management materials.

In this study, we are committed to preparing a material to replace conventional silicone rubber by using calcium alginate and through the ion-exchange curing method between calcium ions and sodium alginate. The further compounded TIM exhibits enhanced thermal conductivity, with negligible oil-bleeding from the matrix under pressure and optimal volatility at high temperatures. This research provides a new strategy for the development of high-performance multifunctional TIMs and addresses the key challenges in thermal management for next-generation electronic and optoelectronic systems.

## 2. Materials and Methods

### 2.1. Materials

All materials utilized in this study were commercially sourced and used without further purification. BN with an average lateral particle size of approximately 60 µm (product code: SP55-C) was obtained from Saint-Gobain, France (Paris, France). Sodium alginate (analytical grade, purity ≥ 90%) was purchased from Macklin Biochemical Technology Co., Ltd. (Shanghai, China). Anhydrous calcium chloride (CaCl_2_) was supplied by Sinopharm Chemical Reagent Co., Ltd. (Beijing, China). For comparative analysis of oil-bleeding and high-temperature volatilization characteristics, a commercially available TIM as TP300, provided by HYMN Advance Materials Technology Co., Ltd. (Shenzhen, China), was selected as a reference benchmark.

### 2.2. Methods

#### 2.2.1. Subsubsection Preparation of Oriented BN/Calcium Alginate Composite in a Hydrogel Matrix

**Figure 1:**
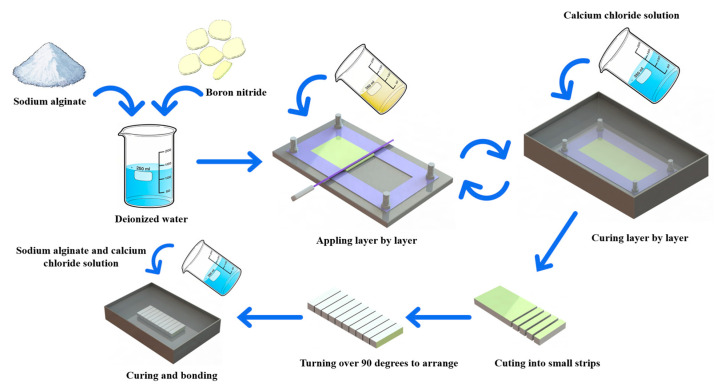
Fabrication of o-BN&Ca-alginate samples.

The preparation process of oriented BN/calcium alginate composite was illustrated in Figure 1. Specifically, 1.5 g of sodium alginate and 60 g of BN were dispersed into 100 mL of deionized water and thoroughly stirred to form a homogeneous BN/sodium alginate slurry. The resulting slurry was then evenly spread onto a thickness-limiting mold and immersed in a pre-prepared calcium chloride (CaCl_2_) solution (3.85 wt.%) for 60 min to complete ion-exchange curing. After the initial curing step, the mold was removed, the limiting plate height adjusted, and a second layer of slurry was applied and cured following the same procedure. This process was repeated until a composite sheet of the desired thickness was obtained.

Subsequently, the cured BN/calcium alginate composite sheet was rinsed with deionized water and dried. The dried sheet was then cut into narrow strips approximately 1.5 mm in width. These strips were flipped so that the cut surfaces faced upward and were then aligned side-by-side. A 1.5 wt.% sodium alginate solution was applied to the top surfaces of the aligned strips, and the assembly was immersed in calcium chloride solution to cure and bond the strips together. Finally, the bonded structure was rinsed and dried, yielding a composite with an oriented distribution of BN fillers within the calcium alginate hydrogel matrix.

#### 2.2.2. Preparation of Randomly Distributed BN/Calcium Alginate Composite in a Hydrogel Matrix

The preparation of BN/calcium alginate composites with randomly distributed BN fillers is relatively straightforward. Initially, 1.5 g of sodium alginate and 60 g of BN were dispersed into 100 mL of deionized water and thoroughly stirred to form a uniform BN/sodium alginate slurry. The slurry was then poured into a mold with a thickness of 1.5 mm, and the mold was immersed in a pre-prepared CaCl_2_ solution containing 3.85 wt.% CaCl_2_. The curing process was allowed to proceed for 24 h to ensure complete ion-exchange crosslinking. Following curing, the resulting composite sheet was removed from the mold, rinsed thoroughly with deionized water, and subsequently dried. The final product is a BN/calcium alginate composite in which BN fillers are randomly distributed throughout the hydrogel matrix.

For the formation mechanism of calcium alginate gel, in aqueous solution, sodium alginate dissociates into Na^+^ and polyanionic chains rich in carboxylate (-COO^−^) groups from guluronic (G) and mannuronic (M) acid residues. Ca^2+^ ions from CaCl_2_ displace Na^+^ through ion exchange, preferentially binding to G-block carboxylates. These divalent cations bridge adjacent alginate chains via chelation, forming a three-dimensional “egg-box” structure stabilized by ionic bonds. The Ca^2+^ ions occupy interstitial sites between parallel G-block helices, generating a porous hydrogel network.

### 2.3. Characterizations

The surface and cross-sectional morphologies of all materials and composites were examined using scanning electron microscopy (SEM; Gemini 300, Zeiss, Oberkochen, Germany). The thermal resistance of the composites was measured using a thermal resistance tester (LW9389, LongWin, Taoyuan, Taiwan), and the tolerance for the equipment testing was 2%. During the LED thermal rise test, the temperature of the LED was monitored using an infrared thermometer (CA-10, Dianytech, Shenzhen, China), and the tolerance for the equipment testing was ±1 °C.

## 3. Results and Discussion

### 3.1. Structural Characterization of Aligned and Non-Aligned BN in Calcium Alginate Matrix

**Figure 2:**
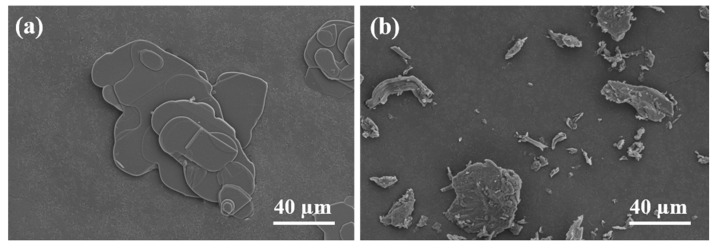
(**a**) SEM morphology of BN; (**b**) SEM morphology of sodium alginate.

Figure 2a presents the scanning electron microscopy (SEM) image of BN filler, clearly exhibiting its characteristic two-dimensional, layered morphology. The BN platelets possess smooth basal planes and sharply defined edges, with a relatively uniform lateral dimension of approximately 60 µm.

In contrast, Figure 2b depicts the microstructure of the sodium alginate, revealing a markedly different and more irregular morphology. The observed topography is amorphous and fragmented, with non-uniform features and significant surface roughness. The irregular edges and jagged or torn morphologies are attributed to two primary factors: (1) the random crosslinking behavior of sodium alginate chains during calcium ion-induced gelation; and (2) the formation of microfractures during the drying process, driven by capillary forces and volumetric contraction of the gel matrix. The resulting fragments display a polydisperse size distribution, with some extending up to 60 µm in their longest dimension. This microstructural heterogeneity underscores the importance of filler alignment in achieving consistent thermal pathways within the composite.

To evaluate the influence of BN orientation on thermal conduction performance within the composite matrix, finite element analysis (FEA) was conducted using COMSOL Multiphysics 6.2. Numerous scholarly studies have validated the efficacy and reference value of employing finite element simulation software to investigate the influence mechanisms of fillers on the performance of TIMs [25,26]. Representative volume element (RVE) models were constructed to simulate the thermal behavior of composites containing either randomly dispersed or vertically aligned BN fillers embedded within a calcium alginate matrix. These simulations were carried out under defined thermal boundary conditions to elucidate the differences in heat transfer characteristics.

Based on the specified filler volume fraction, the software algorithm generates discrete BN fillers within the RVE domain, ensuring either random orientation or vertical alignment. Each generated filler undergoes an overlap validation step; in cases of spatial conflict with existing fillers, regeneration is performed until all fillers are properly positioned without intersection, thus maintaining realistic filler distributions for both random and aligned models. The parameters used in simulation are presented in Table 1. And the contact between the filler and the matrix is a perfect contact without interfacial thermal resistance.

**Figure 3:**
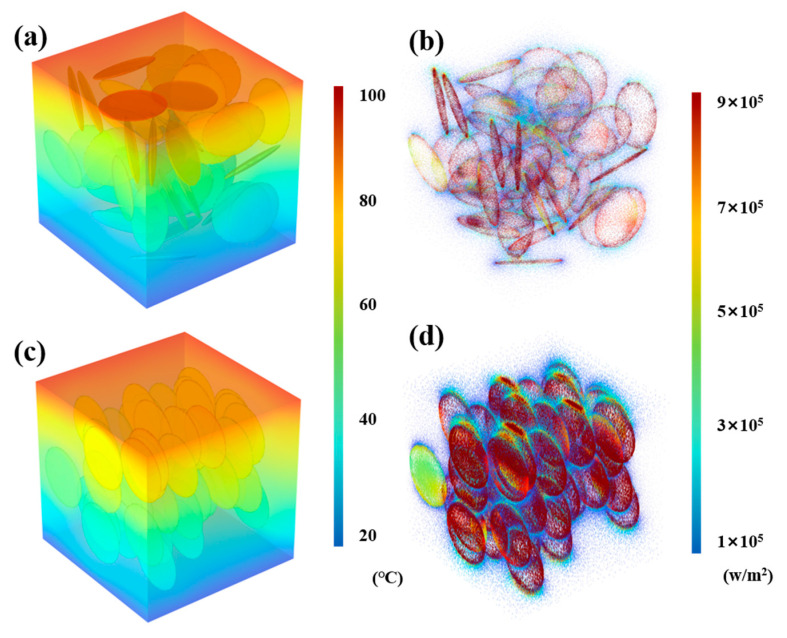
(**a**) Temperature distribution in the RVE model with randomly distributed BN; (**b**) heat flux distribution in the RVE model with randomly distributed BN; (**c**) temperature distribution in the RVE model with vertically aligned BN; and (**d**) heat flux distribution in the RVE model with vertically aligned BN.

Thermal simulations reveal a stark contrast in heat transfer behavior between the two composite configurations. As shown in Figure 3a, the temperature distribution within the RVE model of the randomly dispersed composite (r-BN&Ca-alginate) under a thermal gradient of 100 °C (top boundary) and 20 °C (bottom boundary) indicates significant temperature variation along the heat transfer axis. This steep temperature gradient is indicative of poor thermal conductivity, attributed to the absence of continuous thermally conductive pathways.

Correspondingly, the heat flux distribution depicted in Figure 3b further highlights the inefficiency of the randomly distributed system. The disordered filler network results in low heat flux density and limited directional heat flow, suggesting that thermal energy is primarily confined within the polymer matrix rather than being efficiently conducted through the thermally conductive BN fillers, which is consistent with the conclusion drawn by Zhang et al. [27].

In contrast, the aligned composite model (o-BN&Ca-alginate), shown in Figure 3c, exhibits a more uniform and linear temperature gradient across the RVE, indicative of improved thermal conductivity. This is corroborated by the heat flux analysis in Figure 3d, which demonstrates significantly elevated heat flux density and intensity along the direction of the vertically aligned BN fillers. The alignment facilitates the formation of continuous thermally conductive pathways, enabling efficient heat transmission from the high-temperature to low-temperature boundaries.

These results conclusively demonstrate that the vertical alignment of BN fillers within the calcium alginate matrix significantly enhances thermal transport by establishing anisotropic conduction channels. The formation of such pathways reduces thermal resistance. It improves both the rate and efficiency of heat dissipation, underscoring the importance of filler orientation in the design of high-performance TIMs.

**Figure 4:**
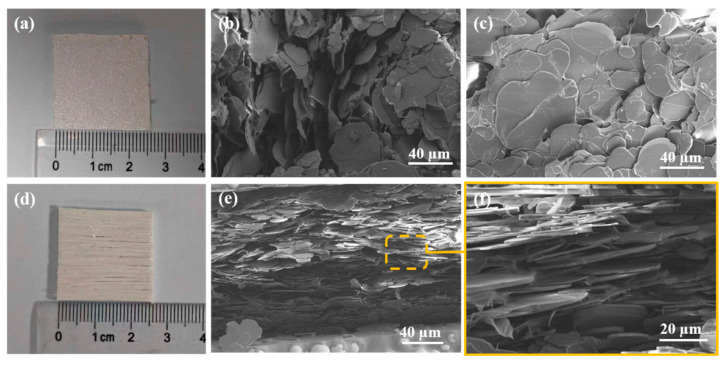
(**a**) r-BN&Ca-alginate sample; (**b**) cross-sectional SEM image of the r-BN&Ca-alginate sample; (**c**) surface SEM image of the o-BN&Ca-alginate sample; (**d**) o-BN&Ca-alginate sample; (**e**) cross-sectional SEM image of the o-BN&Ca-alginate sample; and (**f**) high-magnification cross-sectional SEM image of the o-BN&Ca-alginate sample.

Following the preparation procedures outlined in Figure 1, two types of composite samples were fabricated: a randomly distributed BN/calcium alginate composite (r-BN&Ca-alginate), as shown in Figure 4a, and a vertically aligned BN/calcium alginate composite (o-BN&Ca-alginate), as shown in Figure 4d. Subsequent surface and cross-sectional analyses were performed to evaluate and compare the alignment characteristics of the BN fillers in the respective composites.

Figure 4b displays the cross-sectional scanning electron microscopy (SEM) image of the r-BN&Ca-alginate composite. The BN fillers are randomly dispersed throughout the matrix, with no discernible orientation pattern. Both the basal planes and edges of the BN flakes are visible, indicating an isotropic and disordered distribution of fillers.

In contrast, the surface and cross-sectional morphologies of the o-BN&Ca-alginate composite, presented in Figure 4c and Figure 4e, respectively, exhibit distinct structural order. The surface morphology is notably smooth, and BN flakes are clearly arranged in a planar configuration, demonstrating successful horizontal alignment. The cross-sectional SEM image reveals that only the edges of the BN platelets are visible, suggesting that the flakes are uniformly embedded in a vertical orientation relative to the surface. This observation is further supported by the high-magnification image in Figure 4f, where individual BN platelets can be seen inserted perpendicularly into the matrix with consistent spacing and thickness. The regular, densely packed structure of the BN fillers confirms that the brush-coating method effectively induced a high degree of alignment within the calcium alginate matrix.

The vertically oriented arrangement promotes intimate interfacial contact and dense stacking between adjacent BN flakes, which is essential for constructing continuous and efficient thermal conduction pathways. These findings reinforce the critical role of filler alignment in enhancing the thermal performance of composites designed for thermal interface applications.

### 3.2. Thermal Conductivity of Random and Aligned BN/Calcium Alginate Composites

The thermal conductivity of TIM is a critical parameter for evaluating their heat dissipation capabilities. To assess and compare the thermal performance of the randomly distributed BN/calcium alginate composite (r-BN&Ca-alginate) and the vertically aligned BN/calcium alginate composite (o-BN&Ca-alginate), thermal conductivity measurements were conducted. These tests also serve to validate the conclusions drawn from finite element simulations regarding the effect of filler alignment on heat transfer efficiency.

**Figure 5:**
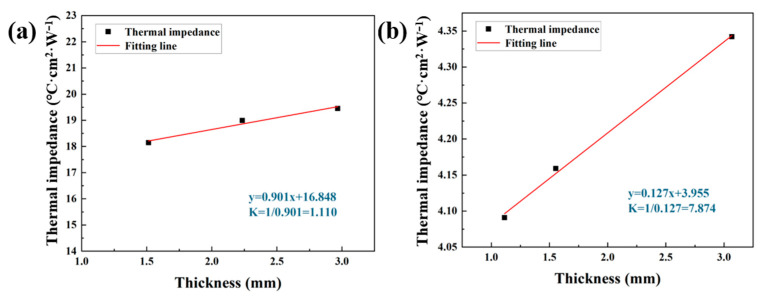
(**a**) Thermal resistance and calculated thermal conductivity of the r-BN&Ca-alginate sample; (**b**) thermal resistance and calculated thermal conductivity of the o-BN&Ca-alginate sample.

Thermal conductivity was measured in accordance with ASTM D5470 using the LW9389 thermal resistance tester. For each composite type, samples with three different thicknesses were prepared, and their thermal resistance values were recorded. As illustrated in Figure 5a,b, thermal conductivity was determined by plotting the measured thermal resistance against sample thickness, with the slope of the linear fit representing the thermal resistance per unit thickness. The inverse of this slope yields the effective thermal conductivity of the material.

Based on the fitted data, the thermal conductivity of the r-BN&Ca-alginate composite was calculated to be 1.11 W·m^−1^·K^−1^, whereas the o-BN&Ca-alginate composite achieved a significantly higher thermal conductivity of 7.87 W·m^−1^·K^−1^. This represents an enhancement of approximately 610% in through-plane thermal conductivity due to the vertical alignment of BN fillers. Such a dramatic improvement highlights the effectiveness of the alignment strategy in establishing continuous heat conduction pathways. Compared to the method used by Niu et al. [28] to obtain TIMs with similar thermal conductivity, the brush-coating method is simpler and more convenient. Moreover, the use of vertically aligned BN not only enhances thermal conductivity but also contributes to a reduced dielectric constant, thereby fulfilling the dual requirements of high thermal performance and low dielectric loss in high-power laser communication devices and advanced optoelectronic systems.

**Figure 6:**
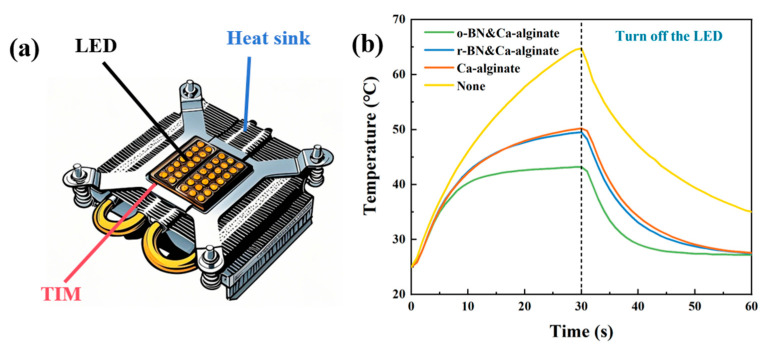
(**a**) Schematic diagram of thermal performance evaluation setup for composites; (**b**) effect of using different composites on the operating temperature of the LED.

In addition to standard thermal conductivity testing, practical thermal management performance was evaluated using a functional LED-heatsink setup, as schematically shown in Figure 6a. Under identical power input and operation duration, the average surface temperature profiles of LEDs—without TIM, with r-BN&Ca-alginate, and with o-BN&Ca-alginate—were recorded and compared. As shown in Figure 6b, the o-BN&Ca-alginate composite exhibited superior heat dissipation performance, as evidenced by the lower steady-state LED temperature during operation. This real-world validation further confirms the substantial thermal management benefits provided by the vertically aligned BN architecture within the calcium alginate matrix.

As illustrated in Figure 6a, when without TIM is applied between the LED and the heatsink, the interfacial thermal resistance increases significantly, impeding the rapid dissipation of heat generated during LED operation. Consequently, the LED temperature rises sharply, reaching 64.8 °C within just 30 s.

When calcium alginate and r-BN&Ca-alginate composites are employed as TIMs, both exhibit moderate improvements in heat dissipation. The r-BN&Ca-alginate sample outperforms calcium alginate slightly, reducing the LED temperature to 49.5 °C after 30 s of operation—a 23.6% decrease compared to the no-TIM condition. This marginal improvement can be attributed to the presence of thermally conductive BN, even though it is randomly dispersed.

Significantly better thermal management performance is observed with the o-BN&Ca-alginate composite. After 30 s of LED operation, the temperature reaches only 43.2 °C, demonstrating the most effective cooling performance. Compared to the no-TIM condition and the r-BN&Ca-alginate sample, the o-BN&Ca-alginate sample achieves temperature reductions of 33.3% and 12.7%, respectively.

These results are consistent with the previously measured thermal conductivity values, confirming that the LED cooling performance directly correlates with the composite’s heat transfer capabilities. Furthermore, while calcium alginate alone possesses low intrinsic thermal conductivity, its application as a TIM still results in a notable temperature reduction relative to the no-TIM condition. This effect can be attributed to its ability to displace air from microscopic interfacial gaps, thereby reducing the overall contact thermal resistance between the LED and the heatsink.

It is also noteworthy that the r-BN&Ca-alginate and o-BN&Ca-alginate composites share identical compositions. The dramatic difference in thermal performance is solely due to the vertical alignment of BN fillers in the o-BN&Ca-alginate, which facilitates more efficient heat transfer pathways.

Additionally, after 30 s of operation, the LED power was switched off, allowing for passive cooling. The temperature of the LED continued to decrease over time due to ongoing heat dissipation. The trend in temperature reduction during the cooling phase mirrored that observed during heating, further validating the superior thermal management capability of the o-BN&Ca-alginate composite.

### 3.3. Oil-Bleeding and Volatilization Behavior of Calcium Alginate-Based TIMs Under Pressure and Elevated Temperature

During long-term service, conventional TIMs are prone to oil-bleeding and volatilization of unreacted organic compounds or low-molecular-weight polymers. These by-products, often residuals from incomplete crosslinking reactions during fabrication, can migrate from the matrix under elevated temperatures and pressure. This phenomenon remains a critical unresolved challenge in TIM development and poses a significant threat to the performance stability of precision optical and electronic components.

**Figure 7:**
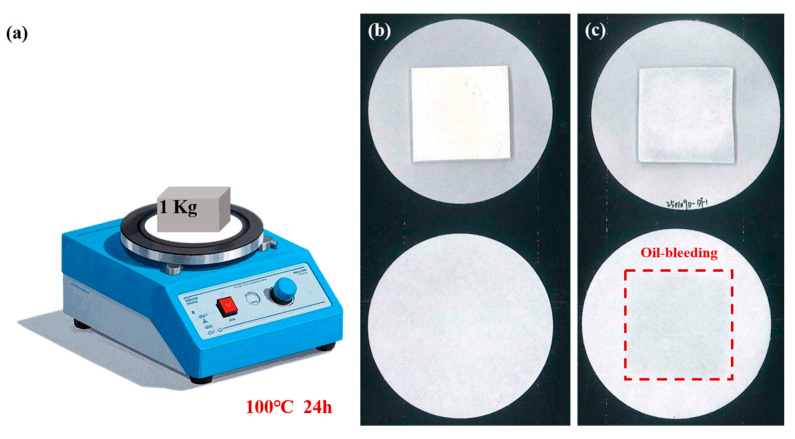
(**a**) Schematic diagram of the oil-bleeding test setup; (**b**) oil-bleeding behavior of the o-BN&Ca-alginate sample; and (**c**) oil-bleeding behavior of the TP300.

To evaluate the oil-bleeding behavior of calcium alginate-based composites under accelerated aging conditions, a comparative study was conducted. Samples of vertically aligned BN/calcium alginate composite (o-BN&Ca-alginate) and a commercially available silicone-based TIM (TP300) were prepared, each with dimensions of 30 mm × 30 mm × 2 mm. As illustrated in Figure 7a, each sample was placed between two filter papers and subjected to a temperature of 100 °C on a heating stage. A 1 kg weight was applied to the top of the filter assembly to simulate mechanical pressure typically encountered in real device environments. The test duration was 24 h, designed to accelerate aging and mimic prolonged operational conditions in electronic and optoelectronic systems.

This experimental setup enabled the assessment of oil-bleeding and condensate formation from the TIMs under combined thermal and mechanical stress, providing a practical evaluation of material stability critical for applications in high-performance thermal management systems.

Figure 7b,c show the filter papers used to evaluate material oil-bleeding from the o-BN&Ca-alginate composite and the commercial silicone-based TIM (TP300), respectively. As observed, the filter paper corresponding to the o-BN&Ca-alginate sample exhibits no visible signs of material oil-bleeding, indicating excellent stability under thermal and mechanical stress. In contrast, the TP300 sample produced prominent stains on the filter paper, suggesting considerable oil-bleeding of matrix materials.

Gravimetric analysis further supports these observations. The mass loss of the o-BN&Ca-alginate sample after testing was only 0.02%, whereas the TP300 sample exhibited a significantly higher mass loss of 0.18%. These results confirm the superior structural and thermal integrity of the calcium alginate-based composite under high-temperature and high-pressure conditions.

**Figure 8:**
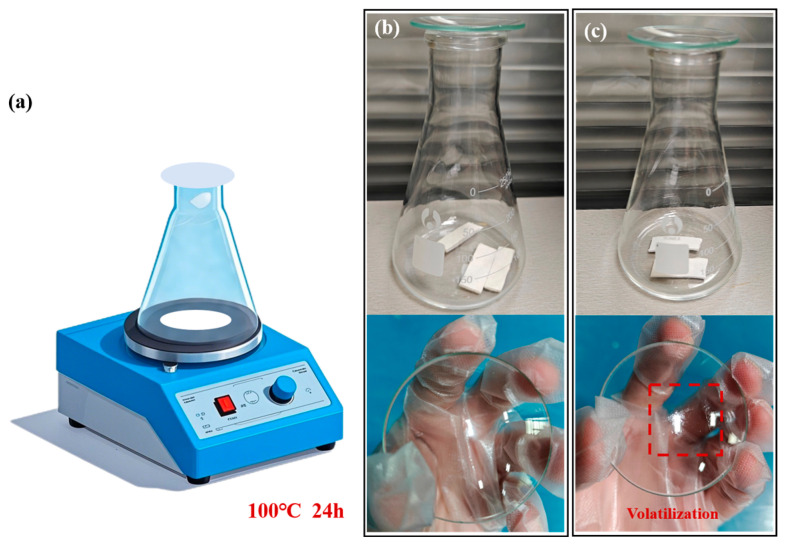
(**a**) Schematic diagram of the volatilization test setup; (**b**) volatilization behavior of the o-BN&Ca-alginate sample; and (**c**) volatilization behavior of the TP300.

To further assess the volatilization behavior of the TIMs, both materials were subjected to a modified condensation test. Samples of o-BN&Ca-alginate and TP300, each weighing 5 g, were individually placed in 150 mL conical flasks and heated at 100 °C for 24 h. A clean glass watch glass was placed at the mouth of each flask to collect any volatile condensates generated during the heating process, as schematically shown in Figure 8a. This setup simulates the accumulation of volatile by-products on sensitive device surfaces in enclosed environments during prolonged operation.

This experimental design allows for a practical evaluation of the volatility of each TIM, offering insight into their long-term reliability in applications where cleanliness and component protection are critical, such as in optical or laser communication systems.

Figure 8b,c present the surfaces of the watch glasses placed at the mouths of conical flasks during the volatilization test, corresponding to the o-BN&Ca-alginate and TP300 samples, respectively. As shown, the glass surface from the o-BN&Ca-alginate test remains clean, with no observable condensate, indicating negligible volatilization of matrix components. In contrast, the TP300 sample produces a distinct oily residue on the watch glass, clearly indicating the presence of volatilized silicone-based compounds.

Gravimetric analysis further confirms these visual findings. The mass loss for the o-BN&Ca-alginate sample was a minimal 0.01%, whereas the TP300 sample exhibited a significantly higher mass loss of 0.26% following the 24-h test at 100 °C. These results demonstrate that the calcium alginate-based composite exhibits excellent thermal and chemical stability, with only a slight mass reduction likely attributable to moisture loss, rather than the volatilization of organic matrix constituents.

This pronounced difference in thermal stability can be attributed to the fundamental differences in matrix structure and intermolecular interactions between the two materials. Calcium alginate forms a robust, ionically crosslinked network through interactions between sodium alginate chains and divalent calcium ions (Ca^2+^), reinforced by hydrogen bonding and ionic interactions. This results in an ordered and tightly bound gel structure with low volatility. In contrast, silicone rubber matrices such as those found in TP300 rely predominantly on van der Waals forces among silicone oil molecules. Unreacted or loosely bound siloxane segments are prone to migration, oil-bleeding, and volatilization under elevated temperature and pressure, leading to undesirable condensate formation.

Together, the oil-bleeding and volatilization analyses confirm that the o-BN&Ca-alginate composite demonstrates superior long-term stability, making it a promising candidate for thermally demanding applications that require cleanliness and material reliability—particularly in precision electronics and optoelectronic systems.

The authors should discuss the results and how they can be interpreted from the perspective of previous studies and of the working hypotheses. The findings and their implications should be discussed in the broadest context possible. Future research directions may also be highlighted.

## 4. Conclusions

In this study, we innovatively adopted the ion exchange curing between sodium alginate and calcium ions as an environmentally friendly alternative to the traditional silicone rubber matrix to prepare high-performance TIMs. Meanwhile, it has achieved the best oil-bleeding and volatility under high temperature and high pressure. The successfully prepared TIM achieved the vertical orientation arrangement of BN fillers in the calcium alginate matrix. The composite obtained by utilizing the excellent thermal conductivity of BN in the vertical direction has a thermal conductivity of 7.87 W·m^−1^·K^−1^.

This method not only resolves the limitations of the thermal stability of traditional silicon-based TIMs but also significantly improves its heat dissipation performance by optimizing the orientation of the filler. On the other hand, it avoids the risks of circuit short circuits, component corrosion, or signal interference caused by the volatilization of organic molecules in traditional silicone-based TIMs. The successful development of this high-performance non-silicone TIM provides a new solution for advancing the next generation of thermal management materials. The results of this study have significant scientific significance and practical value for the application of thermal sensitive technologies such as electronics and optoelectronics.

## Figures and Tables

**Table 1:** Parameters used in simulation.

Material	Density (g·cm^−3^)	Thermal Conductivity (W·m^−1^·K^−1^)	Specific Heat Capacity (J·g^−1^·K^−1^)
Ca-alginate	2.12	0.51	2.0
BN	2.26	56.94	0.8

## Data Availability

The original contributions presented in this study are included in the article. Further inquiries can be directed to the corresponding authors.
